# Measures for the integration of health and social care services for long-term health conditions: a systematic review of reviews

**DOI:** 10.1186/s12913-020-05206-5

**Published:** 2020-04-26

**Authors:** Laura Kelly, Jenny Harlock, Michele Peters, Ray Fitzpatrick, Helen Crocker

**Affiliations:** 1 Nuffield Department of Population Health, University of Oxford, Richard Doll Building, Old Road Campus, Headington, Oxford, OX3 7LF UK; 2 Harris Manchester College, Oxford, OX1 3TD UK; 3 Health Sciences, University of Warwick, Coventry, CV4 7AL UK

**Keywords:** Integrated care, Systematic review, Multi-morbidity, Outcomes, Long-term health conditions, Health and social care integration

## Abstract

**Background:**

As people are living longer with higher incidences of long-term health conditions, there is a move towards greater integration of care, including integration of health and social care services. Integrated care needs to be comprehensively and systematically evaluated if it is to be implemented widely. We performed a systematic review of reviews to identify measures which have been used to assess integrated care across health and social care services for people living with long-term health conditions.

**Methods:**

Four electronic databases (PUBMED; MEDLINE; EMBASE; Cochrane library of systematic reviews) were searched in August 2018 for relevant reviews evaluating the integration of health and social care between 1998 and 2018. Articles were assessed according to apriori eligibility criteria. A data extraction form was utilised to collate the identified measures into five categories.

**Results:**

Of the 18 articles included, system outcomes and process measures were most frequently identified (15 articles each). Patient or carer reported outcomes were identified in 13 articles while health outcomes were reported in 12 articles. Structural measures were reported in nine articles. Challenges to measuring integration included the identification of a wide range of potential impacts of integration, difficulties in comparing findings due to differences in study design and heterogeneity of types of outcomes, and a need for appropriate, robust measurement tools.

**Conclusions:**

Our review revealed no shortage of measures for assessing the structures, processes and outcomes of integrated care. The very large number of available measures and infrequent use of any common set make comparisons between schemes more difficult. The promotion of core measurement sets and stakeholder consultation would advance measurement in this area.

## Background

People are living longer with higher incidences of long-term health conditions now spanning low, middle and high income countries [1]. In response to challenges in rising complex care needs, the integration of care is increasingly appearing on the global agenda with aims of a person-centred approach facilitating both patient and service benefit [2]. Programmes aiming to provide integrated care continue to gather pace, however, further work is needed to identify specific aspects of integration which determine their effectiveness [3–5].

Integration of health and social care services has been a long term policy goal of successive governments within the United Kingdom. Strategies to break down barriers and form partnerships in the provision of health and social care have particularly sought to support people with multiple long-term health conditions [6–8]. The National Health Service (NHS) Long Term Plan further advocates for closer integration of services giving patients more control over the services they receive [9]. Whilst there is clear policy support for integration, the move away from siloed working towards a more collaborative approach to health and social care is challenging. In this context, The Department of Health and Social Care, NHS England and NHS Improvement have recognised the need to clearly define outcomes which they seek to achieve for patients and how they should be assessed [8].

A wide range of definitions and concepts of integrated care exist [10]. Whilst most forms of integration relate to healthcare integration, they can also include integration between healthcare and social care services. NHS England considers integrated care to be:

“person-centred, coordinated, and tailored to the needs and preferences of the individual, their carer and family. It means moving away from episodic care to a more holistic approach to health, care and support needs, that puts the needs and experience of people at the centre of how services are organised and delivered.” [11]*.*

The term integration itself can be further described as the methods, processes and models used to bring about this improved style of coordinated care [12].

The lack of conceptual clarity around integration makes it challenging to identify important outcomes of care and how they should be assessed or measured. It is generally assumed that a more joined up approach to care improves experiences and outcomes for patients and service users through becoming more person centred and reducing system duplication. Although integration has been shown to improve staff and patient perceived quality of care, increase patient satisfaction and improve access to care [13], a more robust evidence base is needed to support these wider assumptions of improvement. A strong evidence base also needs to be extended to assess system and organisational challenges such as cultural differences and data sharing difficulties [14].

There is clearly a drive towards health and social care integration to improve services for patients, however, measuring impact is a challenge [15]. Being able to measure integrated care in a consistent and systematic way is essential for key stakeholders, such as health and social care professionals or policymakers, to advance the design and implementation of a successful integrated health and social care system. Researchers within the integrated care field also need a cohesive measurement strategy so that their research can contribute to an evidence base that can be compared and contrasted meaningfully. With this context in mind, this systematic review sought to identify reviews of effectiveness of integrated care to establish which measures have been used to evaluate the integration of health and social care services for people with long-term conditions. For the purposes of this article, integrated care refers to the integration of health and social care services. We consider ‘social care’ to refer to care that is delivered through a wide range of organisations and professionals within the community, and includes any care and support provided outside traditional health services. For the purposes of this review, the term ‘measure’ is used to refer to any tool used to assess structures, processes or outcomes of care, either from the patients’, carers’, clinicians’ or system perspective.

## Methods

A search was performed across four electronic databases (PUBMED; MEDLINE; EMBASE; Cochrane library of systematic reviews) to identify both systematic and non-systematic reviews of literature regarding the integration of health and social care. See Additional file 1 for search strategy used.

Searches were limited to abstracts and titles only. Additional limits applied were, a publication date from 01/01/1998 to 31/08/2018, to reflect the time period in which integration initiatives have largely been introduced, and restrictions to the adult population. Searches were limited to English language only. Preferred Reporting Items for Systematic Reviews and Meta-Analyses (PRISMA) guidelines were followed [16].

### Study selection and data extraction procedures

Study selection was carried out in two phases. Eligibility was assessed through abstract screening performed by LK and HC against study selection criteria (see Table 1). A third reviewer, MP, screened abstracts where uncertainty of inclusion arose. Full papers were sourced for potentially eligible reviews and underwent further examination by LK and HC to determine if they met the review inclusion and exclusion criteria.

**Table 1:** Study selection criteria

Inclusion criteria	Exclusion criteria
• Adults (> 18 years) • Inclusion of ≥ one measure to evaluate vertical health and social care integration • Focus on population with long-term condition(s)	• Children or adolescents • Article is not a review • Population does not have long-term condition(s) • Focus on integration of health only (i.e. social care not included) • Not published in the English language • Grey literature and commentaries

A data extraction form, which included headings relating to the review’s aims, population, methodology and reported measures, was developed in EXCEL. Data extraction headings were tested by extracting data from three articles (by LK, HC and MP). Data for the remaining articles were extracted by one researcher (LK or HC), but in cases of uncertainty, the second researcher independently extracted data from the same article and results discussed until a consensus was reached.

### Extraction of results

Integrated care measures reported in each review were extracted and collated in a spreadsheet under headings guided by Donabedian’s distinctions between *structures* (where a measure reflects attributes of the healthcare service, for example, the adequacy of facilities and equipment or staff to patient ratios), *processes* (where a measure reflects the way systems work to deliver desired outcomes, for example, patient waiting time) and *outcomes* (where a measure assesses impact on the patient or system, for example reduced length of stay, decreased mortality, and patient experience) [17, 18]. For the purposes of this review, the third component, outcomes, was sub-divided into System Outcomes, Health Outcomes and Patient- and Carer-Reported Outcomes. Once the range of measures were identified, they were extracted and summarised within a spreadsheet. Next, a count of reviews that presented data for structures, processes and outcome (sub-divided into system, health and patient/carer-reported outcomes) headings was produced (see Table 2). The identified measures were summarised, presented in the results section and in Table 3.

**Table 2:** Measures of integration reported in review articles

Author, date	Structures	Processes	Outcomes		
			System outcomes	Health outcomes	Patient- or carer- reported outcomes
Damery et al., 2016 [19]			X		
Bautista et al., 2016 [20]	X	X			
Baxter et al., 2018 [13]		X	X		
Busse and Stahl, 2014 [21]	X	X	X	X	
Cameron et al., 2014 [22]	X	X	X	X	X
Suter et al., 2017 [15]	X	X			X
Laver et al., 2014 [23]		X	X	X	X
Rutten-van Molken et al., 2018 [24]		X	X	X	X
Backhouse, 2017 [25]			X	X	X
Soto et al., 2004 [26]			X	X	X
Tummers et al., 2012 [27]		X	X	X	X
Davies et al., 2011 [28]		X	X	X	X
Eklund et al., 2009 [29]		X	X	X	X
Kirst et al., 2017 [30]	X	X	X		X
Mason et al., 2015 [31]	X	X	X	X	X
Stewart et al., 2013 [32]	X	X	X	X	X
Strandberg-Larsen et al., 2009 [33]	X	X			
Valaitis et al., 2017 [34]	X	X	X	X	X
**Total review articles**	**9**	**15**	**15**	**12**	**13**

**Table 3:** Summary of measures identified in review articles

Measures	Measure sub-group	Topics identified
**Structural**	*Integration ethos: Understanding, appreciation and ‘buy-in’*	Goals/aims aligned [15, 22] Professional roles and responsibilities [22] Management support (Vision, risk management, health and safety, structure, confidence in staff) [22] Perceived systems integration [15] Historical/cultural/contextual issues [15, 22]
	*Communication and information sharing*	Coordination between services and linkages, Inter/intra organisation communication across providers [15, 21, 30, 33, 34], transition policies, efficiency in assessments, case prioritisation, connections with partner organisations, case and care management [15, 22, 30, 33] IT systems and data management [15, 22, 33] IT accessibility to patients [15] Logistic and suitability of information sharing, Co-location [22]
	*Staff*	Team effectiveness, productivity, competency, cohesion, communication, task completion, role performance [15, 23, 30, 34] Teamwork between providers [21, 30] Physician integration in provider collaboration [15, 33]
	*Budget compatibility and resources*	Unified/pooled budgets/integrated management [22, 31] Transfer payments [31] Barriers to financial integration [31] Resource allocation [15]
	*Other organisational*	HR arrangements (e.g. sick leave) [22] Administrative burden [21] Service differentiation [33] Operational and organisational structure integration [15, 33] Clinical integration [33]
	*Other*	Extent of integration (Depth/level/degree of integration) [15, 32] Implementation of integrated delivery, Plan-do integration [33] Care integration and chronic care [20]
**Processes**	*Performance measures*	Quality: Perceived quality, quality standards [13, 15, 21, 28, 31], quality of care transistion [15], quality of care planning, performance management [15] Time spent in emergency/urgent care, length of wait [13], timeliness of assessments [22], timeliness of information transfer [15] Rates of patients leaving insurer [21] Adherence to process measures [27] Improved documentation [28]
	*Patient, family and carer perspectives*	Satisfaction, experience, preferences met, involvement in decision making, incidents of complaints [13, 15, 20, 21, 23, 29–32, 34] Level of empowerment and empathy [15, 30, 32], person centeredness, comprehensive care [20, 24] Personal respect (dignity, confidentiality, autonomy, comfort with care provider) [15, 34], compassionate care, preferred place of death [24] Unmet needs identified [13, 32], meeting needs of patient [22, 34] Carer experiences and satisfaction [23, 31] Quality of interactions [28]
	*Provider experience*	Provider experience, staff satisfaction [21, 34] Work experience [13] Staff stress, role conflict, trust in other team members, frequency of contradictory demands of staff, empowerment, staff wellbeing [15, 22, 34]
	*Coordination and planning*	Cooperation, coordination between providers (patient and provider, provider-patient interaction and transition planning) [15, 20, 21, 33, 34] Coordination following discharge [21] Continuity of care/ continuous [15, 20, 21, 24, 33, 34] Number of patients/existence of care plans, follow ups [15, 21]
**System outcomes**	*Healthcare and social care utilisation: Admissions and length of stay*	Admissions/readmissions (including unscheduled (e.g. due to fall), care home, long term care) [13, 21–23, 25–30], Ambulatory care sensitive hospital admissions [24], Time from event to admission [23], Inappropriate admissions [22], Hospital admissions and nursing home transfers avoided [28] Discharge (including delayed discharge, community discharge, unintended) [22, 23, 31] Emergency and urgent care use [13, 19, 21, 29, 30, 32] Length of stay [13, 19, 21, 23, 26, 27, 29, 30, 32] Entry and retention in primary medical care [26]
	*Healthcare and social care utilisation: Amount of services used*	Number of contacts (including clinicians, case manager, ancillary services) or appointments (GP or outpatient appointments and/or checkups/consultations) [13, 21, 26, 30], Missed appointments [34] Number of checks (clinical measures, e.g. Hb1Ac, BMI, blood pressure, foot exam, kidney function, cholesterol, eye test) [21] Number of home-care hours received per week [22] Numbers of and reasons for referrals [28] Amount of home and health services used (detail not specified) [22, 23, 29, 31] Receipt of regular services [26] Treatment rates [23] Medical services utilisation [26, 34] Follow up and uptake of screening [34] Prescribing (including appropriateness of prescribing and medication administered) [13, 26, 28] Use of volunteer services [32] Community care activity [13] Secondary care activity [13]
	*Accessibility*	Access to other resources [13] Access to services [13] Access to care (for example, to culturally appropriate care, specialty or sub-specialty care) [21, 24, 26, 34]
	*Costs*	Costs [13, 19, 21–24, 26, 29, 30]
	*Other*	Desire to be institutionalized [32], Prevention of premature institutionalisation [34] Financial, employment, and health claims addressed (for example, employment and financial stresses, numbers of mental health patients who applied for disability benefits, behavioural health claims, proportion of patients suffering from mental illness who become insured) [34]. Costs of living at home, justice contacts [24], Vocational status [23]
**Health outcomes**	*Clinical measures*	Mortality [21, 23–25, 27–29, 31, 32], Blood pressure [21], BMI [21], Medication [29], Complications [23], Symptoms (e.g. Head injury [23], pain and other [24]), symptom control [28], Cognition [23], co-morbidities [34]. Adverse events [23] Treatment adherence [26], Adherence rates [23] Condition specific clinical measures (Bowel related problems [28], Percentage healed, mean time to wound healing [28], HbA1c [21], Transmission (mother to child HIV) [26], Problems associated with substance dependence [26], biomarkers for chronic disease [34].
	*Levels of function and disability (clinician rated)*	Function: Health and function [22], function (including physical performance test) [23], physical functioning [24], Functional decline [32], Self-sufficiency [24] Level of disability [23], Activities of daily living/dependency (Barthel Index) [27, 28], Degree of disability or dependence in the daily activities of people who have suffered stroke (Rankin scale) [27] Glasgow Outcome Scale (brain injuries, grouping by degree of recovery) [23]
	*Mental health and behavioural measures (clinician/caregiver rated)*	Mental state (Mini Mental State Examination) [22, 32] Frequency and severity of disruptive behaviours [28] Patient behaviour (Neuropsychiatric Inventory (NPI)) [25] Assessment of change by nursing home staff [28] Neurobehavioral Functioning Inventory [23]
	*Other*	Undefined [31]
**Patient and carer reported outcomes**	*Patient: Health and wellbeing*	Quality of life [22, 25, 27, 29, 30, 34], health-related quality of life [23, 27, 31] Perceived health [29], subjective health [23] Patient outcomes (not specified) [26] Well-being [22] Coping with everyday living [22] QALYs [27]
	*Patient: Physical health*^*a*^	Physical function [25, 27, 29–31], Activities of daily living [23, 28, 29, 34]
	*Patient: Psychological and social factors*	Mood [23, 29] Emotional state [23], worries, concerns and stress [34] Psychological well-being [24] Mental illness symptoms [26], Mental health [34] Depression and anxiety [25, 29, 31], Patient Health Questionnaire (PHQ) [23], Geriatric depression scale [28] Social participation/relationships [24] Social support [25] Resilience [24] Enjoyment of life [24]
	*Patient: Other*	Activation and engagement [24] Autonomy [24], self-efficacy, self-management or empowerment [34], knowledge and understanding of condition [23, 34] Total pain relief (brief pain inventory) [28] Burden of medication [24] Patient cognition/ cognitive function [25, 29, 30] Disease specific measurements (undefined) [29]
	*Carers and family*	Carers’ quality of life [23, 25] Carer outcome (undefined) [23] Caregiver burden [23–25, 29–32] Caregiver mood [25], caregiver strain, depressive symptoms or distress [30, 34] Family relationships [26] Family involvement in care [15] Time spent caring [23]

^a^ Considered patient reported unless specifically reported as clinician reported

This review was not limited to a specific review design but aimed to provide a broad overview of measures used in the evaluation of integrated care. As such we did not systematically evaluate the methodological rigour of each review. The research design is however outlined for each review.

## Results

### Search results and review characteristics

**Fig. 1:**
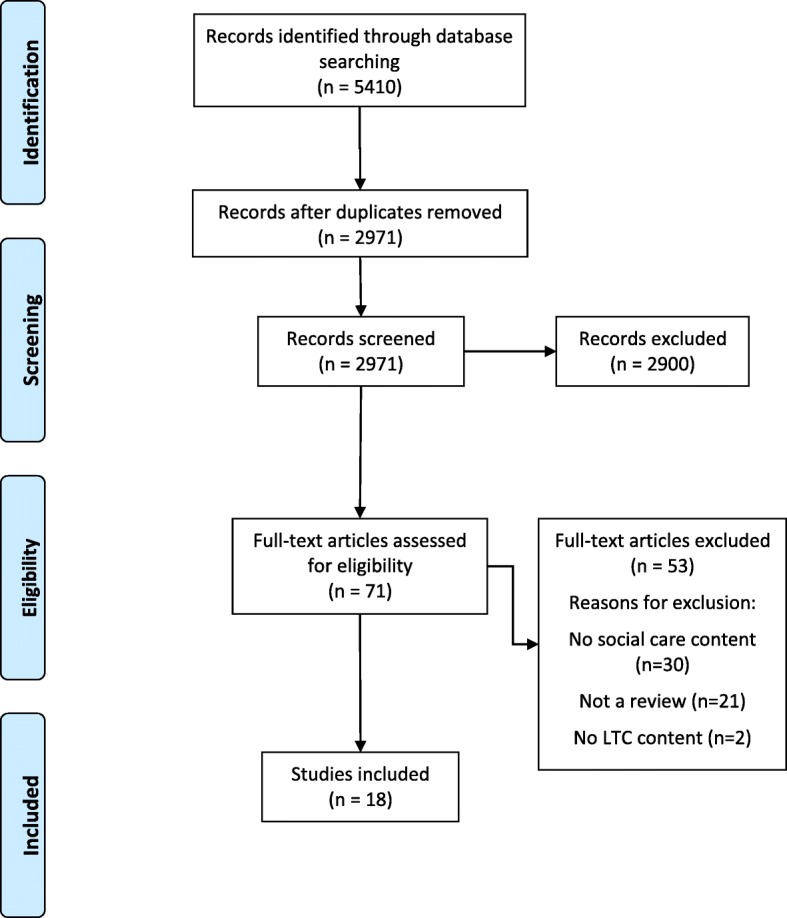
PRISMA flow diagram

Initial searches retrieved 5410 references from electronic databases. After duplicates were removed, 2971 citation records were screened resulting in 71 full text articles for further eligibility assessment. After assessing full text articles, 18 met the inclusion and exclusion criteria (Fig. 1 for PRISMA flow diagram). The main reasons for exclusion among the full text papers included the absence of social care studies in the results or a study design other than a review.

The numbers of articles included in each review article ranged from 9 [29] to 300 [20]. Where reported, reviews reported studies as being conducted in Canada (*n* = 13), Europe (*n* = 13), USA (*n* = 11), Australia and New Zealand (*n* = 8), Asia (*n* = 5) and Africa (*n* = 1). Reviews were described as: umbrella review (*n* = 1), systematic review (*n* = 7), realist review (*n* = 1), narrative review (*n* = 1), literature review (*n* = 3), knowledge synthesis (*n* = 1), meta-analysis (*n* = 1), scoping review (*n* = 1) and other/non-systematic review (*n* = 1). A final article, a development of methodological approaches for evaluating integrated care programmes (*n* = 1), was included as it encompassed a narrative literature review on existing integrated care programme outcomes. Population groups included adults and older people with long-term conditions in a range of health and social care settings.

### Measures used to evaluate health and social care integration

Table 2 provides a summary of the measures identified in the included review articles. The most frequently reported measures were system outcomes and processes, both appearing in 15 articles. Patient- or carer-reported outcomes were identified in 13 articles while health outcomes were reported in 12 articles. The category with the fewest reported measures was structural measures with nine review articles identifying their presence.

### Structural measures

A range of measures for evaluating structural and organisational aspects of integrated care were identified in nine review articles (Table 3). Six articles identified studies which measured aspects of the workforce including staff effectiveness, productivity, competency, cohesion, staff communication, task completion, role performance [15, 23, 30, 34], teamwork between providers [21, 30] and physician integration in provider collaboration [15, 33]. Structural measures which evaluated communication across providers and information sharing were also identified in six review articles. Logistics and suitability of information sharing and co-location [22] were identified as important aspects for evaluation. In addition, coordination and communication between services and organisations [15, 21, 30, 33, 34], transition policies, efficiency in assessments, case prioritisation, connections with partner organisations, case and care management [15, 22, 30, 33] were evaluated. IT systems and data management [15, 22, 33] were measures of successful mechanisms of information sharing. Two articles reported the assessment of the workplace ethos towards the integration of care [15, 22], whilst three outlined measures of budget compatibility and resources [15, 22, 31]. Further organisational measures identified are reported in Table 3.

### Processes

Process measures were identifed in 15 review articles. The most frequently reported categories included performance measures and the measurement of patient, family or carer perspectives of processes of care. Performance measures were included in seven reviews which predominantly consisted of quality-related measures assessing staff and patient perceived quality, quality standards [13, 15, 21, 28, 31], quality of care transistion [15] and, quality of care planning and performance management [15]. Other measures of performance included waiting times for treatment [13], assessement [22] and timeliness of the transfer of information [15]. Further measures of performance are outlined in Table 3.

Thirteen review articles included measures on patient, family, carer and provider perspectives of processes of care. Patients’ perspectives were evaluated through the measurement of patient satisfaction, experience, preferences met, involvement in decision making and, incidents of complaints [13, 15, 20, 21, 23, 29–32, 34]. Measurement of levels of empowerment and empathy [15, 30, 32], person centeredness [20, 24], personal respect (dignity, confidentiality, autonomy, comfort) [15, 34], compassionate care and, preferred place of death [24] were also identified. Assessment of patient needs were reported in four review articles [13, 22, 32, 34]. Measures of carer experiences and satisfaction [23, 31] were identified in two reviews whilst measures of provider experience [13, 15, 21, 22, 34] were identified in five reviews.

### System outcomes

System outcomes were identified in 15 articles mostly identifying measures of health and social care utilisation which broadly covered admissions, discharge and length of stay [13, 19, 21–32]; and measures evaluating the number of services used [13, 21–23, 26, 28–32, 34]. Costs were evaluated in 13 articles [13, 19, 21–24, 26–32]. Measures of accessibility to resources [13], services [13] and culturally appropriate care, specialty or sub-specialty care [21, 24, 26, 34] were reported in five articles. Further details of system outcome measures, including financial, employment, and health claims [34] and vocational status [23] are presented in Table 3.

### Health outcomes

Twelve review articles reported the measurement of health outcomes. The majority of these reported the measurement of clinical health, for example, mortality, blood pressure and various condition specific measures [21, 23–29, 31, 32, 34]. Clinician rated measures of disability and function were reported within six review articles [22–24, 27, 28, 32] whilst five reported the use of proxy (clinician- or caregiver-rated) mental health or behavioural measures [22, 23, 25, 28, 32].

### Patient- and carer-reported outcomes

Thirteen review articles reported the use of patient- and carer-reported outcomes. Patient health and well-being measures, for example, quality of life and coping with everyday life, were identified in nine articles [22, 23, 25–27, 29–31, 34], whilst patient-reported physical health featured in eight [23, 25, 27–31, 34]. Patient-reported psychological and social measures were identified in eight articles, for example, measures of mood, psychological well-being, depression and anxiety [23–26, 28, 29, 31, 34]. Carer-reported measures featured in eight articles and measured areas of carers’ quality of life, mood, distress and burden, and time spent caring [23–26, 29–32, 34]. Family involvement in care was also reported in one article [15]. Further patient-reported outcomes identified (for example, activation and engagement [24]) are reported in Table 3.

### Challenges in measuring the integration of care

There was broad consensus among the reviews that findings regarding the outcomes of integrated care should be treated with caution as current evidence is limited, inconsistent or descriptive [13, 25, 26, 28]. Challenges to measuring the effects of the integration of care included the identification, and appropriate measurement of, a wide range of mechanisms and outcomes which may be impacted across conceptually diverse interventions [22, 28, 33]. Comparisons between studies included within the reviews were considered difficult due to the heterogeneity of outcomes and study design [22, 24, 28]. Few studies reported within study comparison, for example, usual care versus integration of care, making it difficult to determine effectiveness [22]. Where studies included a control group, it was possible to demonstrate impacts on the costs of integrated care comparatively across three countries [21]. More recently, the development of a methodological approach which uses a broad evaluation framework and the use of Multi-Criteria Decision Analysis has shown the potential of cross country intervention comparisons [24]. Here the authors identify and define uniform outcomes for use across 17 programmes and further identify additional programme specific outcomes where relevant.

A number of reviews highlighted the need to ensure appropriate, valid and reliable measures to advance the measurement of integrated care and to support the development of clinical guidelines [20, 26, 33]. One extensive review of structure and process measures, reported a need for higher quality measures with better measurement properties [20]. Whilst they report the majority of measures focusing on care integration and patient-centred care, they highlight a need for less studied constructs, such as care continuity/comprehensive care and care-coordination/case management to become integral in new measures that may need to be developed. In contrast, a knowledge synthesis of domains and tools measuring progress towards integrated care reported the existence of many tools measuring care coordination, patient engagement and team effectiveness or performance, yet few tools for performance management and information systems, alignment of organisational goals and allocation of resources [15].

## Discussion

There is increased recognition of a need for services to bring together a range of professionals and skills from across the health and social care sectors. This integration of care is intended to benefit the service user. Integrated care systems are being implemented; it is vital that assessments of these systems are carried out in a systematic and meaningful way. To do this, appropriate measures of integration need to be established and agreed upon. This review sought to identify measures of integrated care which have been used across both health and social care settings in populations with long-term health conditions.

Eighteen review articles met our inclusion criteria. These articles highlighted an abundance of measures of integration across a spectrum of structures, processes and outcomes. System outcomes and process measures were amongst the most frequently reported measures. This is unsurprising given that system outcome measures largely measured health and social care utilisation and costs, while process measures tended to measure quality, performance and experiences of care. Such system outcomes and process measures are required in the provision of health services [35, 36] and may therefore be considered the most accessible and familiar measures with which to evaluate integrated care in addition to reflecting the priorities of health and social care professionals and decision makers. Despite the drive for integration to provide person-centred and tailored care to benefit the patient, the measurement of patient and carer outcomes were identified slightly less frequently than system outcomes or process measures.

Whilst measures broadly grouped into structural, process and outcome measures, it is of note that we have chosen to include patient experience within the category of process measures. Patient reported experience measures (PREMs) are distinct from patient- or carer-reported outcomes in that, rather than measuring outcomes from the patient or carer perspective, they focus on understanding the patients’ views and experiences of receiving care [37]. Patient Reported Outcome Measures (PROMs) and patient reported experience measures are however frequently used in parallel to gain a picture of both processes and outcomes of care and have been shown to be positively correlated [38, 39]. Patient experience measures appeared frequently in this review, which reflects the emphasis placed upon the coordinated and improved experiences of services in the integration of care services. Despite the debate around the categorisation of the measurement of patient experiences, this review indicates experiences of care are considered an important aspect of patient benefit within the integrated care field.

The findings of this review show a wide range of measures currently being used when assessing integrated care. To facilitate an established body of evidence and advance the evaluation of integrated care programs, standardised measurement sets may help to guide researchers in their choice of tools and study designs. Using a core set of measures would be helpful in the comparison of different schemes and aid the establishment of a clear body of evidence to inform health and social care professionals and policy makers when taking the integration agenda forward. Developing a consensus on core measurement sets however is challenging, not least due to complexity of establishing what an important outcome may be [13]. Guidance may be taken from the vast array of work already carried out to establish core outcome sets for use in clinical research and other healthcare contexts [40]. The development and use of relevant core measurement sets, such as those developed for the Core Outcome Measures in Effectiveness Trials (COMET) initiative to assess effectiveness in trials or the International Consortium for Health Outcomes Measurement (ICHOM) to assess value in health care [41] may help guide and aid comparisons between interventions [42]. Core measurement sets would provide a consensus on important structural measures, process measures and outcomes to measure in future interventions. Measures developed by organisations such as QualityWatch will also be invaluable in the development of a core set to assess policy impact on the quality of care and patient benefit [43]. To get a clearer understanding of measures that are of interest, consultation with stakeholders working in both healthcare and social care settings is vital in steering future recommendations of appropriate measures. In order to improve awareness and support for core measurement sets within integrated care, it would be beneficial to draw on experiences of those in the health outcomes field who advocate for clear channels of communication between key stakeholders [40].

Further exploring relationships between measurement categories may serve to further advance the assessment of integration, for example, how processes impact on outcomes in integrated care settings. Methods which may develop this thinking include the use of logic models which can lay out and unpick understandings of how interventions achieve their intended impacts whilst also identifying factors which underpin the process [44]. Such methods have been explored by the Social Care Institute for Excellence to map enablers, components, outcomes and long-term impacts of integrated care and represent important work in this area [45]. Realist reviews, such as Kirst et al. [30], can also offer insight into the relationships between context, mechanism and action in integrated care programs and identify necessary processes for successful implementation. Unpacking relationships and potential links between structures, processes and, ultimately, outcomes within the integrated care setting is vital in measuring integration success.

Despite a central aim of this review to focus on measures of health and social care integration, the reviews included predominantly focused on healthcare measures. For example, only two reviews identified the inclusion of a significant range of social care measures such as financial, employment and health claims [34]. Measures such as the Adult Social Care Outcomes Toolkit (ASCOT) provide well validated instruments to assess outcomes of social care. Whilst many of the structural and process measures may seem suitable for use in the health care sector, further evidence is needed to evaluate the usefulness of these measures in social care. The large number of healthcare measures may reflect a more developed landscape of quality measures in the health services sector, however it is important to utilise appropriate measures in future evaluations of social care services.

A strength of this study is the rigorous and systematic process of the literature search, carried out in line with PRISMA guidelines [16]. To the best of our knowledge, these findings report a unique overview of all reviews conducted to date which outline measures of health and social care integration. Despite a carefully devised research question and design, a number of challenges were encountered. Firstly, a lack of clarity within articles regarding the inclusion of social services meant it was at times difficult to establish which reviews should be included. Furthermore, on closer inspection of full text articles, many articles which indicated they were reviewing assessments of health and social care integration, had minimal to no social care content in the studies which they identified. Finally, it is possible that ambiguity in how aspects of integration were measured resulted in the misclassification of some measures. These challenges were addressed through assessments of eligibility and the classification of measures by two authors, drawing on a third author where uncertainty arose. Whilst it was outside the scope of this study, it is important to note that some relevant reviews may have been found in reference lists or in grey literature had they been included in our searches.

## Conclusions

An abundance of structural, process and outcome measures to evaluate the integration of health and social care were identified within this review of reviews. However there is no agreement as to a core set of measures, nor was there always clarity about how certain structures, processes and outcomes were measured. Having clearer agreements on which structures, processes and outcomes are important, and specific measures to assess them, would be helpful in evaluating and comparing different schemes and interventions of integration. The use of methods to identify core sets for structures, processes and outcomes of integration interventions would encourage standardisation of measures in evaluations, facilitate reviews and ultimately better support use of evidence by policy makers and service providers. Further exploration of methods, such as logic models or realist reviews, to unpack relationships between components of integrated care may further support a consensus set of measures.

## Supplementary information

**Additional file 1.** Electronic searches.

## Data Availability

All data generated or analysed during this study are included in this published article and its supplementary information files.

## Abbreviations

ASCOT: Adult Social Care Outcomes Toolkit
COMET: Core Outcome Measures in Effectiveness Trials
ICHOM: International Consortium for Health Outcomes Measurement
NHS: National Health Service
PREM: Patient Reported Experience Measure
PRISMA: Preferred Reporting Items for Systematic Reviews and Meta-Analyses
PROM: Patient Reported Outcome Measure
