# Supplementary material for: Measures for the integration of health and social care services for long-term health conditions: a systematic review of reviews
Source: BMC Health Serv Res. 2020 Apr 26;20:358. doi: 10.1186/s12913-020-05206-5 (PMC7183623; doi:10.1186/s12913-020-05206-5)
Supplement: Supplementary file 1 — Additional file 1. Electronic searches. [file 12913_2020_5206_MOESM1_ESM.docx]

# Additional file 1: Electronic searches

The following search strategy was used:

**PubMed search**

(“integrated care”[Title/Abstract] OR “integrated healthcare”[Title/Abstract] OR “health systems integration”[Title/Abstract] OR “care coordination”[Title/Abstract] OR “co-ordinated care”[Title/Abstract] OR “coordinated care”[Title/Abstract] OR “care integration”[Title/Abstract]) AND (Measure*[Title/Abstract] OR “measurement tool”[Title/Abstract] OR tool*[Title/Abstract] OR instrument*[Title/Abstract] OR outcome*[Title/Abstract] OR indicator*[Title/Abstract] OR metric*[Title/Abstract]) AND (review*[Title/Abstract] OR evaluation[Title/Abstract] OR “meta-analysis”[Title/Abstract]) NOT (children[Title/Abstract] OR paediatric[Title/Abstract] OR pediatric[Title/Abstract])

Filters activated: Publication date from 1998/01/01 to 2018/12/31

**Medline and Embase search**

Search terms:

1. integrated care.mp. [mp=title, abstract, original title, name of substance word, subject heading word, floating sub-heading word, keyword heading word, protocol supplementary concept word, rare disease supplementary concept word, unique identifier, synonyms]

2. integrated healthcare.mp. [mp=title, abstract, original title, name of substance word, subject heading word, floating sub-heading word, keyword heading word, protocol supplementary concept word, rare disease supplementary concept word, unique identifier, synonyms]

3. health systems integration.mp. [mp=title, abstract, original title, name of substance word, subject heading word, floating sub-heading word, keyword heading word, protocol supplementary concept word, rare disease supplementary concept word, unique identifier, synonyms]

4. care coordination.mp. [mp=title, abstract, original title, name of substance word, subject heading word, floating sub-heading word, keyword heading word, protocol supplementary concept word, rare disease supplementary concept word, unique identifier, synonyms]

5. co-ordinated care.mp. [mp=title, abstract, original title, name of substance word, subject heading word, floating sub-heading word, keyword heading word, protocol supplementary concept word, rare disease supplementary concept word, unique identifier, synonyms]

6. coordinated care.mp. [mp=title, abstract, original title, name of substance word, subject heading word, floating sub-heading word, keyword heading word, protocol supplementary concept word, rare disease supplementary concept word, unique identifier, synonyms]

7. care integration.mp. [mp=title, abstract, original title, name of substance word, subject heading word, floating sub-heading word, keyword heading word, protocol supplementary concept word, rare disease supplementary concept word, unique identifier, synonyms]

8. 1 or 2 or 3 or 4 or 5 or 6 or 7

9. Measure*.mp. [mp=title, abstract, original title, name of substance word, subject heading word, floating sub-heading word, keyword heading word, protocol supplementary concept word, rare disease supplementary concept word, unique identifier, synonyms]

10. measurement tool.mp. [mp=title, abstract, original title, name of substance word, subject heading word, floating sub-heading word, keyword heading word, protocol supplementary concept word, rare disease supplementary concept word, unique identifier, synonyms]

11. tool*.mp. [mp=title, abstract, original title, name of substance word, subject heading word, floating sub-heading word, keyword heading word, protocol supplementary concept word, rare disease supplementary concept word, unique identifier, synonyms]

12. instrument*.mp. [mp=title, abstract, original title, name of substance word, subject heading word, floating sub-heading word, keyword heading word, protocol supplementary concept word, rare disease supplementary concept word, unique identifier, synonyms]

13. outcome*.mp. [mp=title, abstract, original title, name of substance word, subject heading word, floating sub-heading word, keyword heading word, protocol supplementary concept word, rare disease supplementary concept word, unique identifier, synonyms]

14. indicator*.mp. [mp=title, abstract, original title, name of substance word, subject heading word, floating sub-heading word, keyword heading word, protocol supplementary concept word, rare disease supplementary concept word, unique identifier, synonyms]

15. metric*.mp. [mp=title, abstract, original title, name of substance word, subject heading word, floating sub-heading word, keyword heading word, protocol supplementary concept word, rare disease supplementary concept word, unique identifier, synonyms]

16. 9 or 10 or 11 or 12 or 13 or 14 or 15

17. review*.mp. [mp=title, abstract, original title, name of substance word, subject heading word, floating sub-heading word, keyword heading word, protocol supplementary concept word, rare disease supplementary concept word, unique identifier, synonyms]

18. evaluation.mp. [mp=title, abstract, original title, name of substance word, subject heading word, floating sub-heading word, keyword heading word, protocol supplementary concept word, rare disease supplementary concept word, unique identifier, synonyms]

19. meta-analysis.mp. [mp=title, abstract, original title, name of substance word, subject heading word, floating sub-heading word, keyword heading word, protocol supplementary concept word, rare disease supplementary concept word, unique identifier, synonyms]

20. 17 or 18 or 19

21. 8 and 16 and 20

22. limit 21 to yr="1988 -Current"

23. limit 22 to abstracts

24. children.mp. [mp=title, abstract, original title, name of substance word, subject heading word, floating sub-heading word, keyword heading word, protocol supplementary concept word, rare disease supplementary concept word, unique identifier, synonyms]

25. paediatric.mp. [mp=title, abstract, original title, name of substance word, subject heading word, floating sub-heading word, keyword heading word, protocol supplementary concept word, rare disease supplementary concept word, unique identifier, synonyms]

26. pediatric.mp. [mp=title, abstract, original title, name of substance word, subject heading word, floating sub-heading word, keyword heading word, protocol supplementary concept word, rare disease supplementary concept word, unique identifier, synonyms]

27. 24 or 25 or 26

28. limit 27 to abstracts

29. limit 28 to yr="1998 -Current"

30. 23 not 29

**Cochrane database search**

“integrated care” OR “integrated healthcare” OR “health systems integration” OR “care coordination” OR “co-ordinated care” OR “coordinated care” OR “care integration” in Title Abstract Keyword AND Measure* OR “measurement tool” OR tool* OR instrument* OR outcome* OR indicator* OR metric* in Title Abstract Keyword AND review* OR evaluation OR “meta-analysis” in Title Abstract Keyword NOT children OR paediatric OR pediatric in Title Abstract Keyword - with Cochrane Library publication date between Jan 1998 and Aug 2018 (Word variations have been searched)**'**
